# Supplementary material for: Efficacy and safety of different antidepressants and anticonvulsants in central poststroke pain: A network meta-analysis and systematic review
Source: PLoS One. 2022 Oct 13;17(10):e0276012. doi: 10.1371/journal.pone.0276012 (PMC9560062; doi:10.1371/journal.pone.0276012)
Supplement: S1 Appendix — (DOC) [file pone.0276012.s002.doc]

[**Appendix**](https://www.baidu.com/link?url=Dbhks5TAQEw159BL2OLQ6cofZM5mOXYvTAS9fyQYg8Sq93d2HWXzx-heq-3IkdRTlAPxWclkSODJ_kSUVLToWhW4a1YipsNdCJgpLlCXRte&wd=&eqid=83715cad00028a76000000066317136d)

**Retrieval strategy**

**PUBMED:**

(central poststroke pain[Title/Abstract]) OR (central post-stroke pain[Title/Abstract]) OR (central neuropathic pain after stroke[Title/Abstract]) OR (central neuropathic pain after cerebrovascular disease[Title/Abstract]) OR (central neuropathic pain after cerebral infarction[Title/Abstract]) OR (central neuropathic pain after intracerebral hemorrhage[Title/Abstract]) OR (central poststroke pain[MeSH Terms]) OR (central post-stroke pain[MeSH Terms]) OR (central post stroke pain[Title/Abstract]) OR (central post-stroke pain[MeSH Terms]) OR (thalamic pain[Title/Abstract])

597

**Cochrane Library:**

(central poststroke pain):ti,ab,kw OR (central post-stroke pain):ti,ab,kw OR (central neuropathic pain after stroke):ti,ab,kw OR (central neuropathic pain after cerebrovascular disease):ti,ab,kw OR (central neuropathic pain after cerebral infarction):ti,ab,kw OR (central neuropathic pain after intracerebral hemorrhage):ti,ab,kw OR (central post stroke pain):ti,ab,kw OR (thalamic pain):ti,ab,kw

212

**EMBASE:**

'central poststroke pain':ab,ti OR 'central post-stroke pain':ab,ti OR 'central neuropathic pain after stroke':ab,ti OR 'central neuropathic pain after cerebrovascular disease':ab,ti OR 'central neuropathic pain after cerebral infarction':ab,ti OR 'central neuropathic pain after intracerebral hemorrhage':ab,ti OR 'central post stroke pain':ab,ti OR 'thalamic pain':ab,ti

683

**APA PsycINFO:**

AB central poststroke pain OR AB central post-stroke pain OR AB central neuropathic pain after stroke OR AB central neuropathic pain after cerebrovascular disease OR AB central neuropathic pain after cerebral infarction OR AB central neuropathic pain after intracerebral hemorrhage OR AB central post stroke pain OR AB thalamic pain

237

**CNKI:**

SU =('zu zhong' + 'nao que xue' + 'nao geng si' + 'nao chu xue' + 'zhong feng') AND SU =('zhong shu xing teng tong' + 'shen jing xing tong' + 'zhong shu xing tong' + 'shen jing xing teng tong' + 'shen jing tong ' + 'zhong shu tong' + 'qiu nao tong' )

329

**WANFANG:**

theme:("zu zhong" or "nao que xue" or "nao geng si" or "nao chu xue" or "zhong feng") and theme:("zhong shu xing teng tong" or "zhong shu xing tong" or "shen jing tong" or "shen jing xing tong" or "shen jing xing teng tong" or "zhong shu tong" or "qiu nao tong")

680

**VIP:**

K=("zu zhong" OR "nao que xue" OR "nao geng si" OR "nao chu xue" OR "zhong feng") AND K=("zhong shu xing teng tong" OR "zhong shu xing tong" OR "shen jing tong" OR "shen jing xing tong" OR "shen jing xing teng tong" OR "zhong shu tong" OR "qiu nao tong")

178
