# Supplementary material for: Efficacy and safety of different antidepressants and anticonvulsants in central poststroke pain: A network meta-analysis and systematic review
Source: PLoS One. 2022 Oct 13;17(10):e0276012. doi: 10.1371/journal.pone.0276012 (PMC9560062; doi:10.1371/journal.pone.0276012)
Supplement: Attachment — Submitted filename: Response to Reviewers.doc [file pone.0276012.s005.doc]

Response to editor

1. Please ensure that your manuscript meets PLOS ONE's style requirements, including those for file naming.

**Response:The format has been revised according to your requirements.**

2. In your Data Availability statement, you have not specified where the minimal data set underlying the results described in your manuscript can be found. PLOS defines a study's minimal data set as the underlying data used to reach the conclusions drawn in the manuscript and any additional data required to replicate the reported study findings in their entirety. All PLOS journals require that the minimal data set be made fully available.

**Response: Because this paper is a meta-analysis, all data can be extracted directly from the literature included in this paper. We have uploaded my study’s minimal underlying data set as Supporting Information files.**

3. Please review your reference list to ensure that it is complete and correct.

**Response:We have reviewed my reference.**

Response to reviewers

1. Is the manuscript technically sound, and do the data support the conclusions?

The manuscript must describe a technically sound piece of scientific research with data that supports the conclusions. Experiments must have been conducted rigorously, with appropriate controls, replication, and sample sizes. The conclusions must be drawn appropriately based on the data presented.

Reviewer #1: Yes

Reviewer #2: Partly

**Response: The statistical results in this meta-analysis support this conclusion.**

2. Has the statistical analysis been performed appropriately and rigorously?

Reviewer #1: Yes

Reviewer #2: I Don't Know

**Response:The statistical methods of this meta-analysis are all operated in strict accordance with the guidelines for meta-analysis**

3. Have the authors made all data underlying the findings in their manuscript fully available?

The PLOS Data policy requires authors to make all data underlying the findings described in their manuscript fully available without restriction, with rare exception (please refer to the Data Availability Statement in the manuscript PDF file). The data should be provided as part of the manuscript or its supporting information, or deposited to a public repository. For example, in addition to summary statistics, the data points behind means, medians and variance measures should be available. If there are restrictions on publicly sharing data—e.g. participant privacy or use of data from a third party—those must be specified.

Reviewer #1: Yes

Reviewer #2: Yes

**Response: We have uploaded my study’s minimal underlying data set as Supporting Information files.**

4. Is the manuscript presented in an intelligible fashion and written in standard English?

PLOS ONE does not copyedit accepted manuscripts, so the language in submitted articles must be clear, correct, and unambiguous. Any typographical or grammatical errors should be corrected at revision, so please note any specific errors here.

Reviewer #1: No

Reviewer #2: Yes

**Response: According to your request, I have polished my language to a certain extent.**

5. Review Comments to the Author

Please use the space provided to explain your answers to the questions above. You may also include additional comments for the author, including concerns about dual publication, research ethics, or publication ethics. (Please upload your review as an attachment if it exceeds 20,000 characters)

Reviewer #1: The network methodology has been conducted properly. Yet, the language needs to be revised, especially in the introduction, with a more proper word choice.

e.g. in the sentence " Some studie shave shown that CPSP has a certain latency, which can occur at the latest 18 months" can be rephrased in a more scientific language way.

**Response:According to your request, I have polished my language to a certain extent. We have also revised some words and sentences that are not smooth.**

Reviewer #2: This is a valuable research on treatment of Central post stroke pain. The manuscript is presented in an intelligible fashion and written in standard English except for few spilling mistakes in discussion and conclusion sections. In introduction it will be nice to write in short about pathophysiology of central post stroke pain. What about duration of treatment and its relation to efficacy of drugs? Also, what about the site of lesion in stroke and its relation to efficacy of drugs ?

**Response: We have revised some grammatical problems in our article by AJE. In the introduction, we added the pathogenesis of CPSP, the common disease site, and the commonly used course of treatment and effect of drugs according to your requirements. However, there are very few relevant studies on the relationship between the lesion site of stroke and the efficacy of drugs, so we did not add this part.**

**Some of our major modifications:**

1. **The network meta-analysis registration number is added in the Materials and Methods.**
2. **At the request of the reviewer, some contents were added to the introduction, and three references were added.**

**[3]Leijon, G., Boivie, J., & Johansson, I. (1989). Central poststroke pain: Neurological symptoms and pain characteristics. Pain, 36(1), 13-25**

**[4]Oh H S, Seo W S. A Comprehensive Review of Central Post-Stroke Pain[J]. Pain Management Nursing, 2015, 16(5):804-818**

**[5] Treister AK, Hatch MN, Cramer SC, Chang EY. Demystifying Poststroke Pain: From Etiology to Treatment. PM R. 2017 Jan;9(1):63-75. doi: 10.1016/j.pmrj.2016.05.015. Epub 2016 Jun 16. PMID: 27317916; PMCID: PMC5161714.**

**3.author: Ke-Yu Chen1¶, Ruo-Yang Li2*¶**
